# Transforming Growth Factor-β1 and Laminin-111 Cooperate in the Regulation of Expression of Interleukin-6 and Interleukin-8 in Synovial Fibroblasts

**Published:** 2010-12

**Authors:** Tino Felka, Katrin Warstat, Torsten Kluba, Falk Mittag, Maik Hoberg, Gerd Klein, Wilhelm K. Aicher

**Affiliations:** 1 *Center for Regenerative Biology and Medicine (ZRM), Center for Medical Research, University of Tübingen Medical School, Tübingen, Germany;*; 2 *Department of Orthopaedic Surgery, University of Tübingen Medical Center (UKT), Tübingen, Germany;*; 3 *Department of Orthopaedics and Trauma Surgery, KRI, Technical University, Munich, Germany;*; 4 *Section for Transplanation Immunology and Immunohematology, Center for Medical Research, University of Tübingen, Germany*

**Keywords:** arthritis, inflammation, IL-6, IL-8, TGF-β synovial fibroblast

## Abstract

In a recent study we showed that binding of synovial fibroblasts (SF) to laminin-111 (LM-111) in the presence of TGF-β1 induced a significant production of IL-16. Here we go on to investigate the regulation of IL-6 and IL-8 in SF by LM-111 and TGF-β1. Changes in steady state mRNA levels encoding the interleukins were investigated by quantitative RT-PCR. We screened for interleukin production by a multiplexed immunoarray and quantified it with ELISA. The biological activity of IL-6 and IL-8 was corroborated by B-lymphocyte proliferation and cell migration assays, respectively. Growth of SF on LM-111 in presence of TGF-β1 induced significant mRNA responses for IL-6 (mean 3.72-fold increase, ± 1.6, *p*<0.003) and IL-8 (mean 4.5-fold increase, ± 1.6, *p*<0.001). In the supernatants significantly elevated concentrations of IL-6 (mean 7.9 ± 5 ng/mL, *p*<0.005) and IL-8 (mean 73.0 ng/mL ± 51, *p*<0.05) were detected, and they were shown to be biologically active. Binding to LM-111 in the presence of TGF-β1 activates SF for expression of IL-6 and IL-8 and thus may contribute to synovial inflammation and to infiltration of leukocytes.

## INTRODUCTION

In rheumatoid arthritis (RA), inflammation and infiltration of mononuclear cells contribute to synovial hyperplasia (1). The key regulatory factors for inflammation in RA are TNF-α and IL-1β, but IL-6 or IL-8 contribute to the RA pathology as well (2–5). IL-6 is a key factor for activation of B- and T-cells and for the mobilization of neutrophils (6, 7). IL-8 promotes chemotaxis of neutrophils (8), lymphocytes (9) and mononuclear cells (10).

Laminin-111, previously called laminin-1 (LN-1) or EHS laminin, was detected in the pericellular matrix of articular cartilage (11). Using an anti-EHS-laminin antibody, high expression of laminins has been detected in the lining layer of the synovial membrane of RA patients (12). Activation of synovial fibroblasts (SF) by binding to LM-111 in the presence of TGF-β1 activated the expression of MMP-3, MMP-10 and IL-16 (13, 14). This activation of SF by TGF-β1 and LM-111 works in the absence of TNF-α and IL-1β does not depend on NFκB signalling, and thus may contribute to tissue destruction in the joints of RA patients, even after administration of pharmaceuticals controlling bioactive TNF-α or IL-1β.

High expression of IL-6, IL-8 and IL-16 were measured in RA synovial fluid (15, 16), and the activation of SF by LM-111 in the presence of TGF-β1 resulted in a significant IL-16 expression (14). Therefore, we extended our recent studies and investigated whether LM-111 and TGF-β1 cooperate in regulation of additional RA-associated interleukins in SF, specifically of IL-6 and IL-8.

## METHODS

### Cell culture

SF were isolated from synovial tissue of 17 OA and 16 RA patients as described recently (Table 1) (14). The cells were expanded in DMEM medium (Life Technologies) enriched with ITS (Life Technologies), 10% FCS (Biochrome) and antibiotics (Sigma). This study was approved by the local ethics committee.

**Table 1:** Clinical data of patients included in the study

Diagnosis	Number of patients	Gender of patients	Mean age	Mean CRP	Mean BSR	DMARDs	Steriods	NSAR

RA	16	F9/M7	61.8	0.77	29.1	13/16	10/16	4/16
OA	17	F9/M8	66.5	0.40	14.5	0/17	1/17	12/17

RA, rheumatoid arthritis; OA, osteoarthritis; F, female; M, male; CRP, C-reactive protein; BSR, blood sedimentation rate; DMARDs, disease modifying anti-rheumatic drugs; NSAR, non-steroidal anti-inflammatory drugs.

For activation, SF were incubated in DMEM complete medium in the presence of rhTGF-β1 (10 ng/mL, 24 h, Calbiochem). Cells in medium without TGF-β1 served as controls. In other experiments, cells were incubated for 24 h in flasks coated with LM-111 (BD Biosciences). Uncoated flasks and flasks coated with LM-511/521 (Chemicon) served as controls.

### Transcript analysis

RNA was extracted (RNeasy, Qiagen) and cDNA was generated by oligo-(dT) priming and AMV-reverse transcriptase (Clontech) as described (14). Transcripts were quantified by qRT-PCR utilizing commercially available primers (SearchLC) and normalized to GAPDH and serial dilutions of a recombinant standard (Roche) in each run (17). The results are presented as mean transcript induction index of activated fibroblasts normalized to the mock-treated cells and the recombinant standards.

### Protein analysis

Production of cytokines was detected in SF supernatants by a multiplexed cytokine array technique (Luminex^®^). Cells were activated for 24 h by the addition of 10 ng/mL rhTGF-β1, by binding to coated LM-111 or by binding to LM-111 in the presence of TGF-β1. Controls were incubated in normal tissue culture flasks without stimuli. Supernatants were harvested and pre-cleared by centrifugation (12000 x g, 4°C, 5 min), and aliquots were mixed with dye-loaded microbeads coated with antibodies reactive with the cytokines followed by biotinylated detection antibodies and fluorochrome-labelled detection reagent for quantification (Invitrogen). The cytokine array data were confirmed by ELISA (λscan Bio-Tek) and commercially available kits (GE Health Care).

Biological activity of IL-6 was confirmed by the B9 B-cell hybridoma proliferation assay (a generous gift of Prof. Kolodziej, Berlin). Briefly, supernatants of activated SF (n=8) were added to B9 cells and IL-6-dependent cell proliferation was determined utilizing a modified MTT assay (EZ4U, Biomedica). Complete medium and dilutions of rhIL-6 (GE Health Care) served as controls. Activity of IL-8 was determined by a transmembrane cell mobility assay, in which supernatants of activated cells were added in a modified Boyden chamber and the number of HL-60 cells migrating through the membrane was counted (18). Complete medium and dilutions of rhIL-8 (GE Health Care) served as controls.

### Statistics

The statistical evaluation of the experimental data was performed using a two-sided t-test. The probability values (p) equal to or less than 0.05 (*), 0.01 (**) or 0.001 (***) were considered to be statistically significant and marked in the figures accordingly. The data present the mean values ± standard deviations of individual experiments with cells of n patients (14≥n≥3).

## RESULTS

### Activation of interleukin mRNA expression in synovial fibroblasts

**Figure 1:**
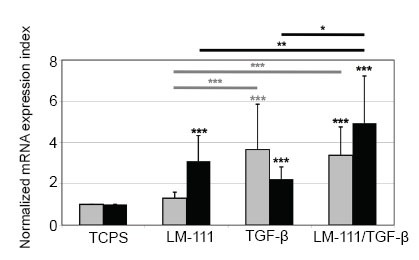
Regulation of expression of interleukins in synovial fibroblasts. Synovial fibroblasts were incubated for 24 h in TCPS flasks (control), in flasks coated with LM-111, in TCPS flasks in presence of 10 ng/ml TGF-β1 or in flasks coated with LM-111 and in addition activated by 10 ng/ml TGF-β1. The induction of mRNA encoding IL-6 (grey bars) and IL-8 (black bars) was investigated by qRT-PCR. Binding to LM-111 induced a moderate IL-8 response (3.1 ± 1.24; *p*<0.001) but no IL-6 response (1.3 ± 0.3, *p*>0.007) in comparison to the TCPS controls. Activation of SF by TGF-β1 induced a moderate and significant IL-6 response (3.6 ± 2.2, *p*<0.001), whereas IL-8 displayed a significant but very low response (2.2 ± 0.5, *p*<0.001) in comparison to TCPS controls. Binding of SF to LM-111 in the presence of TGF-β1 induced IL-6 (3.3 ± 1.4, *p*<0.001) and IL-8 (4.9 ± 2.3, *p*>0.001) significantly. Statistically significant differences among the three experimental groups are marked by crossbars. The data represent the mean transcript amounts ± standard deviations in cDNA of SF from n patients (11<n<14) and are presented as the normalized transcript induction index over mock-treated SF as controls.

Activation of SF by attachment to LM-111 induced a moderate but significant IL-8 mRNA response when compared to SF attached to tissue culture polystyrene (TCPS) flasks. The induction of IL-6 mRNA by LM-111 was low and did not reach significance (induction index ≤2, Fig. 1). Binding of SF to LM-511/521 served as a control but failed to activate the expression of the interleukins investigated (not shown). TGF-β1 induced a moderate but significant IL-6 transcript response and a low but significant IL-8 response (Fig. 1). Activation of SF by attachment to LM-111 in the presence of TGF-β1 induced significant mRNA responses for IL-6 and IL-8 (Fig. 1).

### Production of interleukins by activated synovial fibroblasts

**Figure 2:**
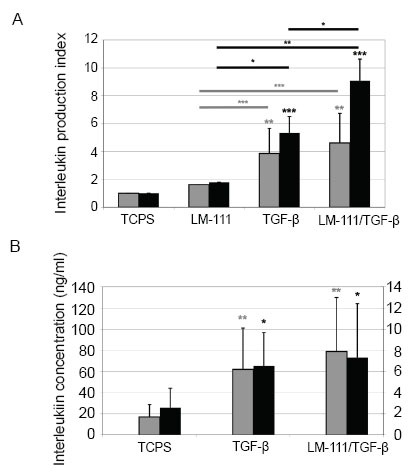
Detection of interleukins in supernatants of synovial fibroblasts. A, SF were incubated for 24 h in uncoated TCPS flasks or in LM-111 coated flasks. We screened for cytokines in the SF supernatants by a multiplexed protein array (Luminex^®^). Growth of SF on LM-111 failed to boost the production of IL-6 (grey bars) and IL-8 (black bars). Activation of SF by TGF-β1 (w/o LM-111) significantly raised the cytokine release of IL-6 (3.9-fold ± 1.8, *p*<0.001) and IL-8 (5.3-fold ± 1.2, *p*<0.02) compared to mock-treated controls (TCPS). Co-activation of SF by TGF-β1 and LM-111 further enhanced the release of IL-6 (4.6-fold ± 2.1, *p*<0.001) and IL-8 (9.1-fold ± 1.56, *p*<0.001). The LM-111 plus TGF-β-induced production of IL-8 was significantly higher than the TGF-β (*p*<0.02) or LM-111- (*p*<0.002) induced responses (black crossbars). The TGF-β- and the LM-111 plus TGF-β- induced production of IL-6 were significantly higher (*p*<0.001 each) than the LM-111- induced response (grey crossbars). Data represent the normalized mean induction index ± standard deviation of interleukin concentrations measured in triplicates over controls (TCPS). B, SF were incubated for 24 h in uncoated TCPS flasks (=control), in TCPS flasks in medium enriched with 10 ng/ml TGF-β1, or in LM-111 coated flasks in medium enriched with 10 ng/ml TGF-β1 as indicated. We measured the concentrations of IL-6 (grey bars, scale to the right) and IL-8 (black bars, scale to the left) in the supernatants by ELISA. In TCPS flasks SF produced spontaneously 1.7 ± 1.1 ng/ml of IL-6 and 25.8 ± 18.4 ng/ml of IL-8. Addition of TGF-β1 significantly stimulated the production of IL-6 (6.2 ng/ml ± 3.9, *p*<0.008) and IL-8 (65.4 ng/ml ± 31.4, *p*<0.02). Co-stimulation of SF by LM-111 and TGF-β1 enhanced the production of IL-6 (7.8 ng/ml ± 5, *p*<0.005) and IL-8 (73 ng/mL ± 51.3, *p*<0.04) to some degree. The data represent the mean values ± standard deviations of supernatants from n≤6 SF from RA and OA patients.

The production of cytokines by activated SF was investigated by a multiplexed array (Fig. 2A). Activation of SF by TGF-β1 significantly raised the cytokine induction index for IL-6 and IL-8. Co-activation of SF by LM-111 and TGF-β1 further enhanced the release of IL-6 and IL-8 (Fig. 2A). The concentrations measured for IL-10 (c_max_<0.5 pg/ml), IL-12 (c_max_<2 pg/ml), and TNF-α (c_max_<0.5 pg/ml) were at or below the detection levels. The maximal IL-1β concentrations were measured at 13 pg/ml (not shown).

The production of IL-6 and IL-8 by activated SF was confirmed by ELISA. The addition of TGF-β1 significantly enhanced the production of IL-6 and IL-8, and co-stimulation of SF by LM-111 and TGF-β1 further enhanced IL-6 and IL-8 production (Fig. 2B). The maximal concentration of IL-1β as determined by ELISA remained close to 10 pg/ml in all supernatants investigated (not shown). In SF from 2 out of 8 donors no IL-8 production was detected, but IL-6 was recorded. The reason for this partial unresponsiveness must be clarified in additional investigations.

### Biological activity of fibroblast-produced IL-6 and IL-8

To further confirm the relevance of our studies we investigated the proliferation of B9 B-cells, which are highly dependent of IL-6. Supernatants from activated SF increased the number of viable B9 cells as determined in an MTT assay by 16% compared to medium as a control (Table 2). The biological activity of IL-8 produced by the activated SF was determined in a cell migration assay. The supernatants of activated SF increased the number of migrating HL60 myeloma cells 2.2-fold in comparison to controls (Table 2). We conclude that the IL-6 and IL-8 produced by SF after this activation were biologically active.

**Table 2:** Investigation of biological activity of IL-6 and IL-8 in supernatants of activated SF

Cytokine	SF supernatant	Neg. control	Pos. control

IL-6	1.16	1	2.19
IL-8	2.2	1	2.3

SF were activated by TGF-β1 and attachment to LM-111 for 24 h. The supernatants were harvested, pre-cleared by centrifugation and the biological activity was determined. IL-6 was detected utilizing the IL-6 dependent proliferation of B9 hybrodima cells, IL-8 by cell migration of HL-60 myeloma cells. DMEM complete medium without addition of cytokines served as negative controls, DMEM complete medium enriched with recombinant IL-6 or IL-8 served as positive controls.

## DISCUSSION

Activation of SF by attachment to LM-111 in presence of TGF-β1 induced an elevated expression of IL-6 and IL-8, without involving IL-1β or TNF-α. The expression of TNF-α and IL-1β is especially elevated during an acute phase of rheumatoid arthritis. In this stage transcription factor NFκB serves as an important switch, regulating a variety of cytokines, proteases and other mediators associated with synovitis in RA, including IL-1β, IL-6 and IL-8 (5, 19). In contrast, the TGF-β1 plus LM-111 dependent activation of SF does not seem to be an important trigger in acute inflammatory processes. But this TGF-β1-dependent pathway may maintain a chronic inflammation independent of or after acute flare, albeit at low or moderate levels. It may also contribute to the activation of SF during or after capture of IL-1β or TNF-α by so-called biologicals, such as anakinra, infliximab, etanercept, adalibumab or alike. The TGF-β1 plus LM-111-activated SF produced 6–8 ng/ml of IL-6 and about 70 ng/ml of IL-8 *in vitro* within 24 h of stimulation. This is within the range reported for IL-6 in RA synovial fluid or plasma (15), and more than reported for IL-8 in RA synovial fluid (16) or plasma (20).

In contrast to IL-16 (14), the regulation of IL-6 or IL-8 by TGF-β1 plus LM-111 did not significantly differ between RA-SF and OA-SF (not shown), suggesting that this mode of regulation of IL-6 and IL-8 is not a specific feature of RA. This finding supports our notion that TGF-β1 and LM-111 may regulate IL-6 and IL-8 expression in SF independently of acute inflammation. Moreover, the RA patients were not in an active stage of flare-up at the time point of surgical treatment. This suggests that inflammation characterized by elevated production of IL-1 or TNF-α are not involved in the regulatory pathways described above.

But TGF-β1 is detected in both, RA and OA synovial fluid (21). Elevated expression of different laminin chains in the synovial membrane is more pronounced in RA than in OA samples, but direct proof for over-expression of the a1- and g1 chains of LM-111 is missing (12). On the other hand, LM-111 was described recently as a component of articular cartilage, which is enriched in the pericellular zone in particular (11). Components of the pericellular matrix of cartilage may be exposed to SF during degenerative processes or after injury, and thus contribute to an inflammatory process fuelled by LM-111 or laminin-derived fragments originating from cartilage.

In summary, we provide evidence that binding of synovial fibroblasts to LM-111 in the presence of TGF-β1 triggers a moderate but significant response of IL-6 and IL-8. This pathway seems not to contribute to the pathology of arthritis during the stages of flare-up and acute inflammation, but may well propagate chronic catabolic processes in a joint, even when IL-1β or TNF-α are not expressed at pathological levels or are captured pharmacologically.
